# Editorial to the Special Issue Sensors and Signal Analysis for Dynamic Measurement in Industrial Process

**DOI:** 10.3390/s23249784

**Published:** 2023-12-12

**Authors:** Yandan Jiang, Manuchehr Soleimani, Guanghui Liang

**Affiliations:** 1 State Key Laboratory of Industrial Control Technology, College of Control Science and Engineering, Zhejiang University, Hangzhou 310027, China; 2 EEE Department, University of Bath, Bath BA2 7AY, UK; m.soleimani@bath.ac.uk; 3 Tianjin Key Laboratory of Process Measurement and Control, School of Electrical and Information Engineering, Tianjin University, Tianjin 300072, China; ghliang@tju.edu.cn

Measurement is the front-end basis of information acquisition. Dynamic measurement is critical for the system design, optimal control, status or safety monitoring, energy conservation and emission reduction in industrial processes. Currently, dynamic measurement is a focal point of study. Increasing attention is being paid to dynamic measurement in industrial processes due to the scientific and technological challenges that it still poses with its complexity, time-varying property and intractable nature. Accurate measurement is essential for the theoretical development and characteristic description of dynamic processes. A metrological approach, where issues such as dynamic calibration, uncertainty reduction and evaluation are particularly considered, can contribute to greatly improving the quality of such measurements. 

This Special Issue “Sensors and Signal Analysis for Dynamic Measurement in Industrial Processes” of *Sensors* seeks to explore new research proposals on this increasingly important topic. The ten accepted papers in this issue cover the latest developments and recent improvements in multiphase flow measurement [1,2,3,4], system calibration and dynamic compensation [5,6], flow boiling monitoring [7], rock tunnel blasting [8], microgravity propellant mass gauging [9] and high-voltage electric field measurement [10]. These works present new sensing techniques and measurement methods, novel sensor design and system development, and advanced signal analysis and processing methods to promote the theoretical development and technical advancement of dynamic measurements in industrial processes. Various kinds of sensors/systems are involved in the studies, including contactless conductivity/impedance detection (CCD/CID) sensors, microwave transmission sensors, tomographic sensors (dual-modality electromagnetic flow tomography (EMFT) and electrical tomography (ET) sensors, and electrical capacitance volume tomography (ECVT) sensors), distributed optical systems, vibration meters and electric field measuring sensors.

In [1], Wang et al. investigate the impedance (real part, imaginary part and amplitude) response characteristics of a CID sensor on slug flow in small channels, where the influence of the slug separation distances on the response characteristics is studied. The experimental results obtained with different slug separation distances in the small channels reveal that the slug separation distance has significant effects on the response characteristics of the CID sensor. For a specified small channel, there is a critical value of the slug separation distance that causes the interference of the impedance signals of two adjacent slugs, and this value is different for different parts of the impedance. The research results can provide useful reference for further parameter measurement and CID sensor development for slug flow.

In [2], Zuo et al. establish theoretical models between the mixture properties of water cut under varying salinity conditions and microwave signals (phase shift and amplitude attenuation) based on a microwave transmission sensor, and they propose an algorithm for determining water cut and salinity simultaneously. An uncertainty analysis is applied to the iteration algorithm under test conditions. With the uncertainty distribution, the measurement error of the proposed method can be predicted. The experimental results show that an accuracy higher than 95% in water cut measurements can be expected under the 0–100% water cut range, and an error of about 10% in water conductivity is achievable under water continuous flow conditions. 

In [3], Arif et al. investigate the feasibility of using the state estimation approach for the dynamic image reconstruction of two-phase oil–water flow based on the dual-modality tomography of electromagnetic flow tomography (EMFT) and electrical tomography (ET). By considering the interactions between the velocity field and phase fraction distributions, the spatio-temporally distributed velocity field and oil phase fraction are estimated using the extended Kalman filter (EKF) and fixed-interval Kalman smoother (FIKS), and, further, the volumetric flow rates of the phases are estimated. The experimental results demonstrate that the state estimation approach is feasible and can improve the dual-modal EMFT-ET imaging of two-phase flow in comparison to stationary imaging methods.

In [4], Sheng et al. develop a new contactless cross-correlation velocity measurement system with a three-electrode construction for the velocity measurement of gas–liquid two-phase flow in small channels based on the CCD technique. The system consists of an upstream/downstream switching unit, an upstream signal processing unit and a downstream signal processing unit. Experiments are carried out to evaluate the measurement accuracy and uncertainty of the system. The maximum relative error of the flow rate measurement is 4.54% for bubble flow with a velocity range of 0.312–0.816 m/s, and 3.70% for slug flow with a velocity range of 0.161–1.250 m/s. 

In [5], Figwer et al. propose a new correction method for the dynamic properties of data acquisition systems by attaching supplementary discrete-time filters. This supplementary discrete time filter, estimating values of delayed samples of the measured signal, is identified in the design stage and attached to the output of the data acquisition system. Four simulated case studies differing in the data acquisition system structure and dynamical properties are designed to validate the effectiveness of the proposed method. The results show that the method improves the accuracy of the data acquisition system, measured via the correction quality index, by a few times up to more than 100.

In [6], Crenna et al. develop and test a method for the dynamic compensation of a piezoelectric accelerometer, which is the outcome of a general probabilistic model of the measurement process. The proposed method has been fully developed and tested only for first-order measurement systems, and the authors extend it to second-order systems and apply it to an accelerometer. Thus, the scalar problem is converted into a vector problem. The effectiveness of the method is tested in both simulations and dedicated experiments. The experimental results show the capability of the method in significantly improving the performance of measurement systems when dynamic effects are more prevalent than additive observation noise. 

In [7], Li et al. develop a new distributed optical measurement system to obtain flow information and to determine the boiling intensity of the flow in a plate-fin heat exchanger (PFHE), which utilizes numerous optical fibers installed on the surface of the PFHE to detect optical signals. By analyzing the fluctuation of the optical signal intensity, the development of flow boiling with different heat fluxes is studied in experiments. The experimental results verify the effectiveness of the measurement system in obtaining useful information on flow boiling. And the research results reveal that boiling development in PFHE comprises four stages with the increase in the heating flux, namely, the unboiling stage, the initiation stage, the boiling developing stage and the fully developed stage.

In [8], Yin et al. conduct a large number of on-site tests and comprehensively compare the vibration signals of digital electronic and nonel detonators in a small section of a rock tunnel construction from the perspective of time, frequency and energy. The research results prove that the delay time error of the detonator can control vibration wave random interference and reduce vibration. It is found that, when using a segmented simultaneous blasting network for excavation in small sections of rock tunnels, nonel detonators may provide more excellent protection to structures than digital electronic detonators, while digital electronic detonators are superior to nonel detonators because of the fragmentation effect on rock.

In [9], Chowdhury et al. investigate a number of mass fraction estimation techniques based on a 12-plate spherical ECVT sensor for propellant mass gauging under micro-gravity conditions. The effect of various positions and shapes of the fuel body on the propellant mass gauging under micro-gravity conditions is studied with an electrical capacitance volume tomography (ECVT) sensor. The difficulties in mass fraction estimation under micro-gravity conditions are addressed, and different fuel shapes and positions that commonly occur under micro-gravity conditions are considered. The comparison results of several curve-fitting-based approaches and a machine-learning-based approach show that the latter offers superior performance in estimating the fuel content. 

In [10], Long et al. focus on the design and implementation of a high-precision inductive wideband electric field measuring sensor (EFMS) to calibrate the nonlinearity of the MV-level lightning impulse voltage measurement system. The influence of the metal shell on the electric field distribution is simulated, and the influence of the electric field non-uniformity coefficient is studied. The results show that the EFMS can be used for the nonlinearity calibration of ultra-high voltage impulse measurement devices, with a proportionality coefficient of 0.05664 V/(kV/m). In mostly uniform and extremely non-uniform fields, the nonlinearity of the EFMS for impulse voltage is less than ±0.25%, and the nonlinearity of the EFMS for power-frequency voltage is less than 0.1%. 

In summary, this Special Issue provides a collection of the latest innovative ideas in the dynamic measurement of industrial processes. The high-quality papers provide useful reference for researchers in energy resources, chemical engineering, heat and mass transfer, aerospace engineering, geotechnical engineering, power systems, etc., and they positively contribute to the development of the related fields.
